# Supplementary material for: Data segmentation based on the local intrinsic dimension
Source: Sci Rep. 2020 Oct 5;10:16449. doi: 10.1038/s41598-020-72222-0 (PMC7536196; doi:10.1038/s41598-020-72222-0)
Supplement: Supplementary file 1 — Supplementary information. [file 41598_2020_72222_MOESM1_ESM.pdf]

Supplementary Information for ‘Data segmentation based on the local intrinsic dimension’

Michele Allegra[1,2], Elena Facco[2], Francesco Denti[3], Alessandro Laio[2,4,*], and Antonietta Mira[5,6,+]

[1]Institut de Neurosciences de la Timone UMR 7289, Aix Marseille Université, CNRS, 13385 Marseille, France
[2]Scuola Internazionale Superiore di Studi Avanzati, Trieste, Italy
[3]University of California, Irvine, California, U.S.A.
[4]International Centre for Theoretical Physics, Trieste, Italy
[5]Università della Svizzera italiana, Lugano, Switzwerland
[6]Università dell'Insubria, Como, Italy
[*]laio@sissa.it
[+]antonietta.mira@usi.ch

Enforcing neighborhood uniformity. In our model, we wish to obtain well separated manifolds. We do not wish to impose this condition in the form of a rigid constraint, since in real cases regions with different IDs are not completely separated, but only as a “soft constraint”, privileging configurations of z such that the first neighbors of each point are preferentially assigned to the same manifold. In a Bayesian framework, this means that given that j is among the first neighbors of i, the probability that $z_i = z_j$ is increased. Consider the *neighbor matrix* $\mathcal{N}_{ij}^{(q)}$ defined as:

$$\mathcal{N}_{ij}^{(q)} = \begin{cases} 1 & \text{if } j \neq i \text{ is among the first } q \text{ neighbors of } i \\ 0 & \text{otherwise, including } i = j \end{cases} \tag{S1}$$

Intuitively, we would like to impose

$$P_{post}(z_i = z_j | \mathcal{N}_{ij}^{(q)} = 1, \mu, p) > P_{post}(z_i = z_j | \mathcal{N}_{ij}^{(q)} = 0, \mu, p). \tag{S2}$$

However, Eq. S2 is a relation between posterior probabilities, hence it cannot be directly embedded in the likelihood. What we can specify in the likelihood is the probability of observing the data $\mathcal{N}_{ij}^{(q)}$, given an assignment z of the points. The way to enforce Eq. S2 is assuming that *the first neighbors of each point are preferentially points of the same manifold*. Consider the i-th row of the neighbor matrix, $\mathcal{N}_i^{(q)} \equiv \{\mathcal{N}_{ij}^{(q)}, j = 1, \ldots, N\}$. $\mathcal{N}_i^{(q)}$ is a vector containing q ones and $N-q$ zeros. Without any assumption, all configurations of q zeros and $N-q$ ones are equally likely. Instead, we assume that neighbors are preferentially points from the same manifold. Formally, we assume that neighbors are selected forn the points of the same manifold with probability ξ and from a different manifold with probability $1-\xi$, with $\xi > 1/2$. Correspondingly, we introduce a new term in the likelihood:

$$\mathcal{L}(\mathcal{N}_i^{(q)} | z) = \frac{\xi^{n_i^{in}(z)} (1-\xi)^{q - n_i^{in}(z)}}{\mathcal{Z}(\xi, N_{z_i})}, \tag{S3}$$

where

$$n_i^{in}(z) = \sum_j \mathcal{N}_{ij}^{(q)} \mathbb{I}_{z_j = z_i} \tag{S4}$$

is the number of neighbors of i sampled from the same manifold, and

$$q - n_i^{in}(z) = \sum_j \mathcal{N}_{ij}^{(q)} \mathbb{I}_{z_j \neq z_i} \tag{S5}$$

is the number of neighbors of i sampled from a different manifold. Function $\mathscr{Z}$ is a normalization factor that depends on ξ:

$$\mathscr{Z}(\xi, N_{z_i}) = \sum_{\{\mathscr{N}_i^{(q)}\}} \xi^{n_i^{in}(z)}(1-\xi)^{q-n_i^{in}(z)}. \tag{S6}$$

and can be expressed in a compact way as

$$\mathscr{Z}(\xi, N_{z_i}) = (1-\xi)^q \binom{N-N_{z_i}}{q} {}_2F_1(-q, 1-N_{z_i}, N-N_{z_i}-q, \frac{\xi}{1-\xi}), \tag{S7}$$

where ${}_2F_1(a,b,c,x)$ is the hypergeometric function. The derivation of this expression and the details about the likelihood term in (S3) are presented below. By considering all points i, we obtain the global likelihood

$$\mathscr{L}(\mathscr{N}^{(q)}|z,\xi) = \prod_i \mathscr{L}(\mathscr{N}_i^{(q)}|z,\xi) = \prod_k \frac{\xi^{n_k^{in}}(1-\xi)^{qN_k-n_k^{in}}}{\mathscr{Z}(\xi,N_k)^{N_k}} \tag{S8}$$

where

$$n_k^{in} = \sum_{ij} \mathscr{N}_{ij}^{(q)} \mathbb{I}_{z_i=k} \mathbb{I}_{z_j=k} \tag{S9}$$

is the total number of "internal" neighbors of points from manifold k and

$$n_k^{out} = \sum_{ij} \mathscr{N}_{ij}^{(q)} \mathbb{I}_{z_i=k}(1-\mathbb{I}_{z_j=k}) = qN_k - n_k^{in} \tag{S10}$$

is the total number of "external" neighbors of points from k. Note that since $\mathscr{Z}$ depends on i only through the hidden variables z we are able to split the product into K components.
With this additional term in the likelihood, we obtain

$$\frac{P_{post}(z_i = z_j|\mathscr{N}_{ij}^{(q)} = 1, \mu, p)}{P_{post}(z_i = z_j|\mu, p)} = \frac{\xi}{1-\xi} > 1/2.$$

Derivation of the neighborhood uniformity term. With reference to $\mathcal{N}_i^{(q)}$, without any assumption, all configurations containing q zeros and $N-q$ ones are equally likely. It is easy to compute the number of such configurations. The problem is analogous to the problem of selecting q balls from a box containing $N-1$ balls: we have to choose q neighbors among $N-1$ points, point i being excluded. The number of possible choices is $\binom{N-1}{q}$. Hence, all configurations of $\mathcal{N}_i^{(q)}$ being equally likely we would have

$$\mathcal{L}(\mathcal{N}_i^{(q)}|z) = \binom{N-1}{q}^{-1}, \qquad \forall i.$$

Instead, we assume that the neighbors of a point are preferentially points from the same manifold. Formally, we assume that neighbors are selected with probability ξ among the N_{z_i} points assigned to the same manifold of i, and with probability $1-\xi$ among the $N-N_{z_i}$ points assigned to a different manifold. Here $\xi > 1/2$, so that configurations with neighbors assigned to the same manifold are more likely. Now, the problem is analogous to the problem where we have to select q balls from two boxes, a black box containing N_b balls and a white one containing N_w balls. Before selecting each ball, we choose the box, the black one with probability ξ and the white one with probability $1-\xi$. Clearly, the probability of a choice with n_b black and $q-n_b$ white balls is then proportional to $\xi^{n_b}(1-\xi)^{q-n_b}$. For a given n_b, the number of possible choices of balls is

$$\binom{N_b}{n_b}\binom{N_w}{q-n_b}$$

One can easily verify that $\sum_{n_b=0}^{q}\binom{N_b}{n_b}\binom{N_w}{q-n_b} = \binom{N_b+N_w}{q}$. The probability of a given choice is then

$$\frac{\xi^{n_b}(1-\xi)^{q-n_b}}{\mathcal{Z}}$$

where $\mathcal{Z} = \sum_{n_b=0}^{q}\binom{N_b}{n_b}\binom{N_w}{q-n_b}\xi^{n_b}(1-\xi)^{q-n_b}$. By using the formula (Abramowitz and Stegun, 15.4.1)

$${}_2F_1(-m,b,c,z) = \sum_{n=0}^{m}(-)^n\binom{m}{n}\frac{(b)_n}{(c)_n}z^n$$

where $(a)_n = a(a+1)\ldots(a+n-1)$ is the Pochammer symbol and doing some simple algebra, $\mathcal{Z}$ can be compactly expressed as

$$\mathcal{Z} = (1-\xi)^q\binom{N_w}{q}{}_2F_1(-q,-N_b,N_w-q,\frac{\xi}{1-\xi}).$$

Replacing N_b with $N_{z_i}-1$ (the number of points assigned to the same manifold as i, excluding i), N_w with $N-N_{z_i}$ (the number of points assigned to a different manifold), and n_b with n_i^{in}, we obtain the likelihood of a given configuration of $\mathcal{N}_i^{(q)}$ as

$$\mathcal{L}(\mathcal{N}_i^{(q)}|z,\xi) = \frac{\xi^{n_i^{in}(z)}(1-\xi)^{q-n_i^{in}(z)}}{(1-\xi)^q\binom{N-N_{z_i}}{q}{}_2F_1(-q,1-N_{z_i},N-N_{z_i}-q,\frac{\xi}{1-\xi})}.$$

Choice of the free parameters. In order to find a good configuration for the parameters (q, ξ), we perform tests with

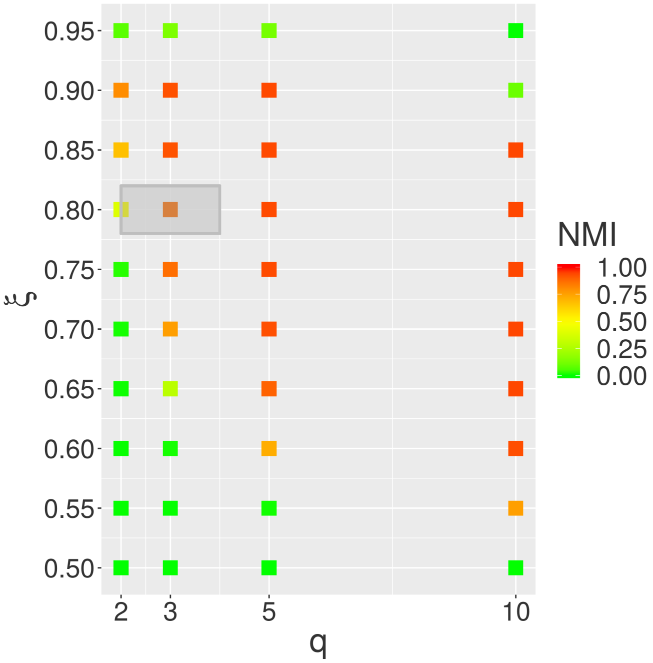

(a) Two Manifolds (linear)

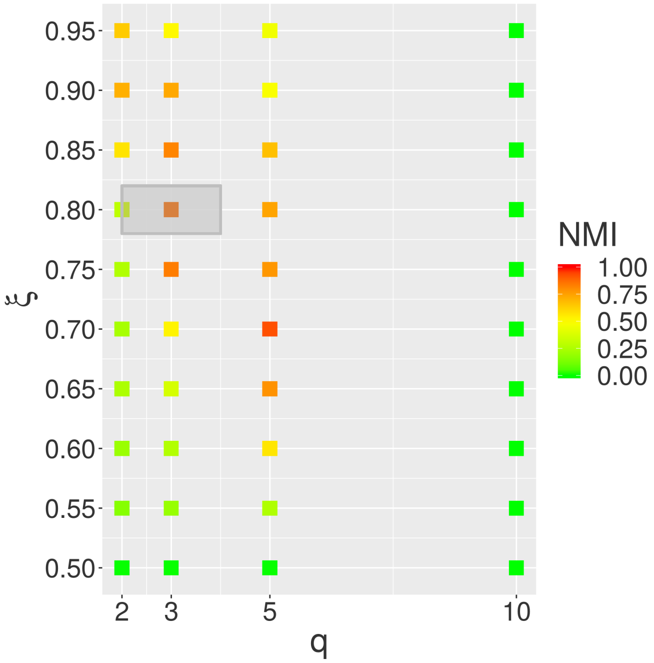

(b) Five Manifolds (linear)

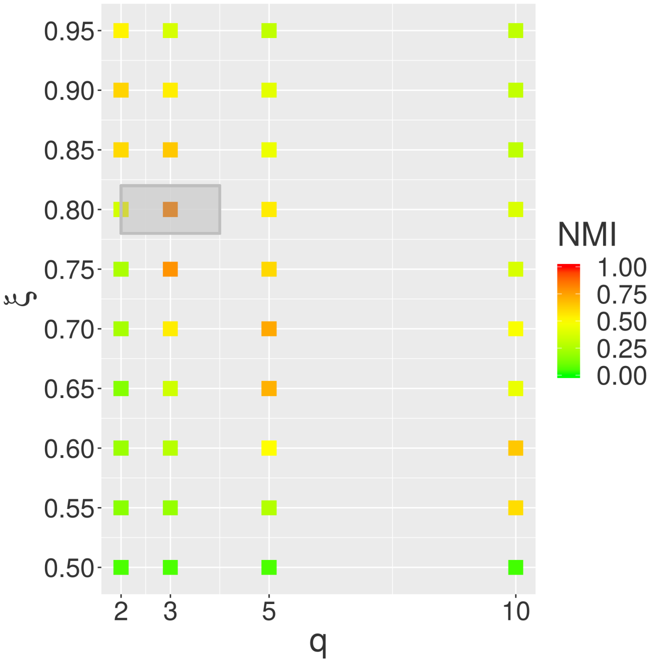

(c) Five Manifolds (curved)

Figure S1. Choice of free parameters. We computed the NMI between the estimated and true assignment of points for all the artificial datasets used for validation, for different valus of q and ξ. a) two manifolds of dimension 4 and 5, with a Gaussian density of points b) five manifolds of dimension $1, 2, 4, 5, 9$, with a Gaussian density of points c) five manifolds of dimension $1, 2, 4, 5, 9$, with a Gaussian density of points, embedded on a 1-dimensional circle, a 2-dimensional torus, a 4-dimensional Swiss roll, a 5-dimensional sphere and a 9-dimensional sphere. In all cases, we performed 10^5 iterations of the Gibbs sampling, and repeated the sampling $M = 10$ times starting from different random configurations of the parameters. We considered the sampling with the highest maximum average of the log-posterior value

several values of $q \in \{2,3,\ldots,10\}$ and $\xi \in [0.5,1)$ for all the artificial datasets used for validation: i) two manifolds of dimension 4 and 5, with a Gaussian density of points; ii) five manifolds of dimension $1,2,4,5,9$, with a Gaussian density of points; iii) five manifolds of dimension $1,2,4,5,9$, with a Gaussian density of points, embedded on a 1-dimensional circle, a 2-dimensional torus, a 4-dimensional Swiss roll, a 5-dimensional sphere and a 9-dimensional sphere.

The crucial figure of merit to assess the performance of the method is the normalized mutual information (NMI) between the estimated and the true assignment of z, which measures the quality of the assignment of points to manifolds. Indeed, once the manifolds are correctly separated, the problem is essentially reduced to a dimension estimation within the single manifolds (which is successfully solved by TWO-NN). In Fig. S1 we show the NMI as a function of (q,ξ). In all cases, we performed 10^5 iterations of the Gibbs sampling and repeated the sampling $M = 10$ times starting from different random configurations of the parameters. We kept the sampling with the highest maximum average of the log-posterior value.

For all values of q, the MI first increases and then decreases with ξ. This can be expected based on the following considerations. When ξ is close to 0.5, as we discussed above, the method cannot discriminate between different manifolds. When ξ is increased, the posterior distribution starts to prefer configurations that approximately satisfy the neighborhood homogeneity constraint. For sufficiently high ξ, the posterior distribution is sharply peaked at the configuration that optimally satisfies this constraint; correspondingly, if the Gibbs sampler can explore the parameter space exhaustively, it will eventually find this peaked region and remain trapped there. Hence, the NMI achieves average values close to 1. However, for ξ close to 1, the posterior distribution is very likely to also have pronounced local maxima. Therefore, depending on the initial configuration, the sampler may remain trapped in one of them. Hence, one can observe a drop in the NMI. In general, these sampling issues can be worsened when q is increased since the local maxima become more and more pronounced. In principle, this problem may be dealt with by resorting to well established enhanced sampling techniques. For simplicity, in the present work, we prefer to verify that there is a region of the parameter space where the results appear optimal and restrict to these regions for subsequent analyses.

In general, $q = 2$ yields poor results ($NMI \lesssim 0.4$) in all cases. This means that is general $q = 2$ is too low to effectively enforce the uniformity constraint. Analogously, $q = 10$ too high yields poor results ($NMI \lesssim 0.5$), except in the simple case of two manifolds. There are two independent reasons for this behavior. First, $q = 10$ can lead to sharp peaks in the posterior, and hence to sampling issues; second, $q = 10$ gives issues in the case of intersecting manifolds, as it enforces uniformity on too large a scale. Good results ($NMI \gtrsim 0.75$) are found for $q = 2, 0.75 \leq \xi \leq, 0.80, 0.85$ and for $q = 5, 0.65 \leq \xi \leq 0.70$. Based on these findings, we identify the optimal “working point" of the method at $q = 3, \xi = 0.8$, which yields $NMI > 0.80$) for all data sets considered.

Convergence time as a function of sample size.

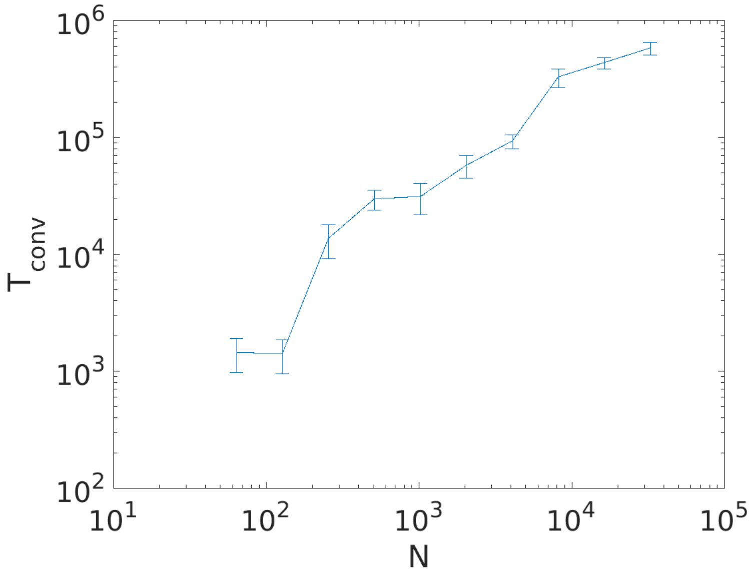

Figure S2. Convergence time T_{conv} of the Gibbs sampling for N points sampled from two manifolds of dimension $d_1 = 9$ and $d_2 = 4$. We repeated the estimation of T_{conv} for $M = 20$ independent samples. The lines connect the average value over the M samples. Standard error bars are reported as well. The scaling of T_{conv} with N is approximately linear.

In order to test the speed of convergence of the Gibbs sampling as a function of the number of points N, we have generated random data sampled from two manifolds of dimension $d_1 = 9$ and $d_2 = 4$. As in Results, $N/2$ points are sampled from a multivariate Gaussian with a variance matrix given by $1/d_i$, $i = 1,2$, times the identity matrix of proper dimension. The two manifolds are embedded in a space with dimension corresponding to the higher dimension d_2, with their centers at a distance of 0.5 standard deviations. We consider different sample sizes $N = 2^i, \{i = 6, 15\}$. In order to test convergence, we use the criterion in[1]. Given independent samples Y_t (with $t = 1,\dots,T$ and T the total number of samples), and given a function $f_t = f(Y_t)$, convergence can be verified from the behavior of the cumulative sums

$$S_t = \sum_{\tau=t_0+1}^{t} (f_\tau - \mu) \tag{S11}$$

where $\mu = \frac{1}{T-t_0}\sum_{\tau=t_0+1}^{T} f_\tau$, and $t_0 \geq 1$ is any time, usually chosen as higher to the putative burn-in time. At convergence, the behavior of F_t is highly non-smooth; In particular, consider the indicator variable

$$e_t = \begin{cases} 1 \text{ if } S_t > S_{t-1} \text{ and } S_t < S_{t-1} \\ 1 \text{ if } S_t < S_{t-1} \text{ and } S_t > S_{t-1} \\ 0 \text{ otherwise} \end{cases} \tag{S12}$$

which indicates whether S_t has a local maximum/minimum at t. At convergence, approximately half of the points should be local minima or maxima. Formally, e_t behaves as a Bernoulli variable $\mathscr{B}(1/2)$, and consequently

$$E_t = \sum_{\tau=t_0+1}^{t-1} e_\tau \tag{S13}$$

behaves as a binomial variable $B(t - t_0 - 1, 1/2)$. Therefore, one should have

$$1/2 - Z_{\alpha/2}/\sqrt{t - t_0 - 1} \leq E_t \leq 1/2 + Z_{\alpha/2}/\sqrt{t - t_0 - 1} \tag{S14}$$

for at least $(1-\alpha)\%$ of the time (for large t, the binomial will be approximately normal). In other words, when convergence is reached, E_t should fluctuate around $1/2$ with a variance compatible with that of a binomial variable.
To adopt this test to the data, we studied the point-wise dimension estimates d_{1t} and d_{2t}. We subsampled the d_{it} at $t = 1, \theta, 2\theta, \dots$

with $\theta = 10$ to ensure independence of the samples. Let D_{1t} and D_{2t} be the resulting samples. We used $f_{1\tau} = d_{1t}$ and $f_{2\tau} = d_{2t}$ and first verified whether the corresponding E_{1t}, E_{2t} (Eq. S13) converged to $1/2$. While an excessive variance around $1/2$ is a sign of residual non-stationarity, failure of E_t to approach the value $1/2$ for increasing t is a likely sign of the non-independence of the samples used[1]. Therefore, if E_{it} did not approach to $1/2$, we increased the subsampling at $\theta = 20, \theta = 50, \theta = 100, \theta = 200, \theta = 500$ until $\langle E_{it} \rangle \simeq 1/2$ for large t. We divided the D_{1t} and D_{2t} in non-overlapping windows of 20 successive points and checked whether E_{it} was within the bounds (S14) with $\alpha = 0.05$ for 95% of the times (at 19 times out of 20) within each window. We then defined as convergence time T_{conv} the time corresponding to the first window where both E_{1t}, E_{2t} were satisfying the criterion.
For each dataset size N, we repeated the assessment of T_{conv} for $M = 20$ independent Gibbs samplings. The results are shown in Fig. S2: one can see that the convergence time increases roughly linearly with data size N.

Restricting the analysis to points with no common first and second neighbor.

Dataset	N_0	N	d_i	NMI
2 Gaussians, $d_1 = 5, d_2 = 4$	2000	357	$d_1 = 5.5,\ d_2 = 3.9$	0.95
2 Gaussians, $d_1 = 6, d_2 = 4$	2000	360	$d_1 = 6.2,\ d_2 = 4.1$	0.93
2 Gaussians, $d_1 = 7, d_2 = 4$	2000	355	$d_1 = 6.5,\ d_2 = 4.3$	0.93
2 Gaussians, $d_1 = 8, d_2 = 4$	2000	365	$d_1 = 9.1,\ d_2 = 3.9$	0.96
5 Gaussians (linear), $d_1 = 1, d_2 = 2, d_3 = 4, d_4 = 5, d_5 = 9$	5000	895	$d_1 = 0.9, d_2 = 1.7, d_3 = 3.2, d_4 = 4.4, d_5 = 9.4$	0.88
5 Gaussians (curved), $d_1 = 1, d_2 = 2, d_3 = 4, d_4 = 5, d_5 = 9$	5000	894	$d_1 = 0.9, d_2 = 1.7, d_3 = 3.2, d_4 = 4.4, d_5 = 9.4$	0.61

Table S1. Independent points. We repeated the analysius of artifical data sets, using only indpedendent points with non-overlapping first and second neighbor For each scenario we show the number of points in the original data set (N_0), the number of points upon restriction N, the estimated value of the d_k, and NMI between the assignment and the ground truth. All results were obtained with $\xi = 0.8, q = 3$, repeating the sampling $M = 10$ times, and considering the sampling with the highest average log-posterior.

Compustat variables used.

#	Variable	#	Variable	#	Variable
1	Acquisitions	12	Liabilities - Total	23	Interest and Related Expense - Total
2	Assets - Total	13	Net Income (Loss)	24	Goodwill
3	Capital Expenditures	14	Operating Income Before Depreciation	25	Intangible Assets - Total
4	Cash	15	Property Plant and Equipment - Total (Net)	26	Pretax Income
5	Common Shares Outstanding	16	Purchase of Common and Preferred Stock	27	Pretax Income - Foreign
6	Common/Ordinary Shareholders	17	Sales/Turnover (Net)	28	Investment and Advances - Equity
7	Debt in Current Liabilities - Total	18	Stockholders Equity - Parent	29	Investment and Advances - Other
8	Long-Term Debt - Total	19	Income Taxes Paid	30	Increase in Investments
9	Cash Dividends (Cash Flow)	20	Research and Development Expense	31	Sale of Investments
10	Earnings Before Interest and Taxes	21	Price Close - Annual - Fiscal		
11	Employees	22	Preferred/Preference Stock (Capital) - Total		

Table S2. **Compustat variables used**

References

1. Brooks, S. P. Quantitative convergence assessment for markov chain monte carlo via cusums. *Stat. Comput.* **8**, 267–274 (1998).
